# DNA damage and repair in peripheral blood mononuclear cells after internal ex vivo irradiation of patient blood with ^131^I

**DOI:** 10.1007/s00259-021-05605-8

**Published:** 2021-11-13

**Authors:** S. Schumann, H. Scherthan, K. Pfestroff, S. Schoof, A. Pfestroff, P. Hartrampf, N. Hasenauer, A. K. Buck, M. Luster, M. Port, M. Lassmann, U. Eberlein

**Affiliations:** 1 Department of Nuclear Medicine, University of Würzburg, Würzburg, Germany; 2 Bundeswehr Institute of Radiobiology affiliated to the University of Ulm, Munich, Germany; 3 Department of Nuclear Medicine, Philipps University Marburg, Marburg, Germany

**Keywords:** DNA damage repair, Radioiodine therapy, γ-h2ax, 53BP1

## Abstract

**Aim:**

The aim of this study was to provide a systematic approach to characterize DNA damage induction and repair in isolated peripheral blood mononuclear cells (PBMCs) after internal ex vivo irradiation with [^131^I]NaI. In this approach, we tried to mimic ex vivo the irradiation of patient blood in the first hours after radioiodine therapy.

**Material and methods:**

Blood of 33 patients of two centres was collected immediately before radioiodine therapy of differentiated thyroid cancer (DTC) and split into two samples. One sample served as non-irradiated control. The second sample was exposed to ionizing radiation by adding 1 ml of [^131^I]NaI solution to 7 ml of blood, followed by incubation at 37 °C for 1 h. PBMCs of both samples were isolated, split in three parts each and (i) fixed in 70% ethanol and stored at − 20 °C directly (0 h) after irradiation, (ii) after 4 h and (iii) 24 h after irradiation and culture in RPMI medium. After immunofluorescence staining microscopically visible co-localizing γ-H2AX + 53BP1 foci were scored in 100 cells per sample as biomarkers for radiation-induced double-strand breaks (DSBs).

**Results:**

Thirty-two of 33 blood samples could be analysed. The mean absorbed dose to the blood in all irradiated samples was 50.1 ± 2.3 mGy. For all time points (0 h, 4 h, 24 h), the average number of γ-H2AX + 53BP1 foci per cell was significantly different when compared to baseline and the other time points. The average number of radiation-induced foci (RIF) per cell after irradiation was 0.72 ± 0.16 at *t* = 0 h, 0.26 ± 0.09 at *t* = 4 h and 0.04 ± 0.09 at *t* = 24 h. A monoexponential fit of the mean values of the three time points provided a decay rate of 0.25 ± 0.05 h^−1^, which is in good agreement with data obtained from external irradiation with γ- or X-rays.

**Conclusion:**

This study provides novel data about the ex vivo DSB repair in internally irradiated PBMCs of patients before radionuclide therapy. Our findings show, in a large patient sample, that efficient repair occurs after internal irradiation with 50 mGy absorbed dose, and that the induction and repair rate after ^131^I exposure is comparable to that of external irradiation with γ- or X-rays.

## Introduction

As stated in a recent position paper of the European Association of Nuclear Medicine (EANM) [1], studying the induction and repair of radiation-induced DNA damage is of high interest for clinical applications of ionizing radiation as it may differ in comparison to external beam radiotherapy (e.g. see [2–4]).

For the detection of DNA double-strand breaks (DSBs) in the low-dose range, the biomarkers γ-H2AX and 53BP1 are widely used. Radiation induces DSBs that in turn lead to the rapid ATM-dependent phosphorylation of the histone H2 variant H2AX, then called γ-H2AX [5–7]. DBSs also recruit the damage sensor 53BP1 to the surrounding chromatin domain [8, 9] where it co-localizes with γ-H2AX [10] to form microscopically visible foci. 53BP1 recruitment also influences DNA damage repair pathway choice and contributes to the repair of DSBs in heterochromatin [11, 12]. Radiation-induced DSBs can be visualized and quantified by microscopically visible DNA damage protein foci that display both γ-H2AX and 53BP1 [13–15]. DSB foci disappear by 53BP1 dissociation and γ-H2AX dephosphorylation after DSB repair has been completed [11, 16].

Ex vivo irradiations offer the possibility to mimic the induction of DNA damage during radionuclide therapies in patients under defined conditions, i.e. at defined absorbed doses and fixed irradiation times. Previously published ex vivo studies of ionizing radiation-induced DSB formation indicate a linear relationship between the number of microscopically visible γ-H2AX and 53BP1 foci and the absorbed dose to the blood after internal ex vivo exposure of peripheral blood mononuclear cells (PBMCs) [4, 14, 17]. However, quantitative data on the repair after ex vivo internal irradiation with β-emitting radionuclides have not been published so far. Until now, ex vivo repair studies have largely been performed after external irradiation by X-rays or γ-rays [18–26] and have generally involved acute irradiation with absorbed doses of > 100 mGy [20, 21, 23–25] to lymphocytes [21, 25], to tumour and other cell lines [23, 26] or fibroblasts [20, 22, 24].

Therefore, the aim of this study was to analyse the induction and repair of DSBs in isolated PBMCs after an internal ex vivo irradiation of whole blood with [^131^I]NaI. In order to mimic the irradiation of patient blood during the first hours after radioiodine therapy, blood samples of patients with differentiated thyroid cancer (DTC) were drawn directly before their first radioiodine therapy and irradiated with a nominal absorbed dose to the blood of 50 mGy, which matches the absorbed dose in the first hours after therapy start [3]. To achieve comparable dose rates, an irradiation period of 1 h was chosen. DSB repair was then investigated over the following 24 h.

This study is a substudy within the European Horizon2020-funded MEDIRAD project (www.medirad-project.eu), a project on investigating the implications of medical low-dose radiation exposure. One of the work packages is looking into the effects of low doses to non-target organs and tissues in patients with differentiated thyroid cancer (DTC) during their first radioiodine therapy.

## Material and methods

### Patient recruitment and ethics statements

For the ex vivo study of DSB damage induction and repair, 33 low-risk patients with DTC, who underwent their first radioiodine therapy for thyroid ablation at the Department of Nuclear Medicine, University of Würzburg (UKW), and the Department of Nuclear Medicine, Philips University Marburg (UKM), according to the respective local standard of care, were included. The main inclusion criteria were the following:

- Total thyroidectomy performed within a maximum of 6–8 weeks before radioiodine treatment
- Histological evidence for low-risk differentiated thyroid carcinoma (DTC)
- No distant metastases

The common study protocol was presented to the local ethics committees at the Medical Faculty of the University of Würzburg (UKW) and Marburg (UKM) and approved. All procedures performed in this study involving human participants were in accordance with the ethical standards of the respective ethics committees (UKW: Az. 246/18, UKM: Az. 83/19) and with the principles of the 1964 Declaration of Helsinki and its later amendments or comparable ethical standards. Informed consent was obtained from all individual participants included in the study.

Patients’ blood withdrawals were performed in the Departments of Nuclear Medicine of the University Hospitals Würzburg (UKW) and Marburg (UKM) by experienced physicians of the department prior to radioiodine therapy. The samples were anonymized for further processing.

### Blood sampling, irradiation and isolation of PBMCs

Prior to activity administration, blood samples were taken from each patient and either not-treated (non-irradiated control) or irradiated with ^131^I ex vivo. Samples were processed according to a custom protocol outlined below, anonymized and sent to the Bundeswehr Institute of Radiobiology in Munich for further processing and quantification of the DSB damage.

The procedure of the ex vivo irradiation was adapted from the protocol reported by Eberlein et al. [14]. Briefly, two 7-ml blood samples were drawn from each patient using Li-Heparin blood collecting tubes (S-Monovette®; Sarstedt) before the radioiodine therapy. One of the samples was internally irradiated by addition of [^131^I]NaI solution: [^131^I]NaI (GE Healthcare) was diluted with phosphate-buffered saline (PBS) so that the concentration was 3.5 MBq per ml. The blood sample was then supplemented with 1 ml of this [^131^I]NaI solution followed by incubation at 37 °C for 1 h to reach an absorbed dose to the blood of 50 mGy. To ensure a uniform irradiation of the blood, samples were incubated on a roller-mixer. The second 7-ml blood sample remained non-irradiated and served as a control, i.e. for determination of background DSB foci numbers. For both samples, PBMCs were isolated by transferring the blood into 8-ml CPT Vacutainer tubes (BD) and centrifuged for 20 min at 1500 g according to the manufacturer’s instructions, followed by two washes in PBS. The isolated cells of each of the two blood samples were split in three aliquots and fixed in an ice-cold 70% ethanol solution directly (*d* = 0 h) or 4 h or 24 h after culture at 37 °C in RPMI medium containing HEPES (Life Technologies) to allow for DNA repair. This resulted in six samples for each patient: three non-irradiated control samples (0-d, 0-4 h, 0-24 h) and three samples irradiated with 50 mGy (50 mGy-d, 50 mGy-4 h, 50 mGy-24 h). Thus, both induction of DNA damage and progression of DNA repair could be compared at different time points. EthOH fixed cells were stored at − 20 °C before shipping to the Bundeswehr Institute of Radiobiology in Munich, Germany, for γ-H2AX + 53BP1 analysis.

### Activity quantification and absorbed dose calculation

^131^I is a β-emitter with a half-life of 8.023 days and a mean β-energy of 181 keV. The γ-emission with the highest probability occurs at 364.5 keV with a decay probability of 81.2% [27].

The *S* value for the blood self-irradiation (${S}_{blood\leftarrow blood}$) for ^131^I was determined using the data published by Hänscheid et al. [28]. The authors used Monte Carlo simulations to determine the energy deposition in blood vessels of different sizes for some radionuclides commonly used in nuclear medicine. This data was adapted to the geometry of our incubation tubes with an inner radius of *r* = 7.2 mm, resulting in ${S}_{blood\leftarrow blood }=3.18\cdot {10}^{-11} \text{Gy }{\text{s}}^{-1}{\text{B}}{\text{q}}^{-1}{\text{ml}}$. With this, the dose coefficient for 1-h irradiation was calculated to 114.16 mGy MBq^−1^ ml. Therefore, an activity of 3.5 MBq was needed to irradiate a volume of 8 ml, resulting in an absorbed dose of 50 mGy.

For an exact quantification of the blood activity concentration in the incubation tubes, an aliquot of 100 µl was taken after incubation and the sample was measured in a high-purity germanium detector (Canberra). The counting efficacy of the detector was ascertained by measuring several NIST- and NPL-traceable standards. All measurements were decay corrected to the start time of the measurement.

### Immunofluorescent staining and evaluation of DNA damage

The ethanol-fixed cells were subjected to cyto-centrifugation followed by immunofluorescent staining to detect DNA damage-associated protein accumulation as microscopic foci at DNA double-strand break sites as described by Ahmed et al. [29]. Primary antibodies against γ-H2AX (Mouse anti-γ-H2AX; Merck) and 53BP1 (Rabbit anti-53BP1; Novus) were applied and detected with secondary goat anti-mouse Alexa-488 (Mobitec) and donkey anti-rabbit Cy3-labeled antibodies (Dianova). The number of radiation-induced DNA damage and repair protein foci was analysed by the same experienced investigator (HS) in 100 PBMC nuclei per sample by manual counting directly in a Zeiss Axioimager 2i fluorescence microscope of the ISIS fluorescence imaging system (MetaSystems) equipped with green and red double band pass filters (AHF Analysentechnik). Images were recorded at 630 × magnification with a Plan-Apochromat 63 × /1.40 oil lens.

To determine the number of radiation-induced foci per cell (RIF), the baseline focus values of each sample and time point (0-d, 0-4 h, 0-24 h) was determined. RIF are, for this study, defined as the difference between the number of foci per cell of the irradiated sample at the three time points 0 h, 4 h and 24 h (50 mGy-d, 50 mGy-4 h, 50 mGy-24 h) and the respective baseline value of the identical non-irradiated sample at the same time point.

### Data analysis

The DNA damage repair is described by a single exponential function with an unrepairable component in analogy to a proposal by Lobachevsky et al. [30]:

1 $$N\left(t\right)={N}_{0}({\left(1-Q\right)e}^{-Rt}+Q)$$

*N*(t): number of RIF per cell at time *t*

*N*_0_: maximum number of RIF per cell

*Q*: the fraction of unrepaired RIF per cell

R: repair rate (h^−1^)

As data were sampled for each patient for three time points after irradiation only, the baseline value *Q* had to be set to zero, thus assuming complete repair, for deriving the individual repair rate for the time-dependent decrease of the average number of RIF.

### Statistical analysis

For data analysis and statistical evaluation, Origin (OriginPro 2019, Origin Lab Corporation) was used. To test whether data were distributed normally, the Shapiro–Wilk test was conducted. For comparing data sets, Wilcoxon signed-rank test for not normal distributed data was used and the paired sample *t* test was used for normal distributed data. Results were considered statistically significant for *p* < 0.05.

## Results

### Patients

For this ex vivo study, 20 UKW patients and 13 UMR patients were included. The patients’ pre-therapeutic blood samples, taken at UMR, were shipped immediately after blood withdrawal to UKW for further processing. Overall, 198 sub-samples (six for each patient), of which 99 were irradiated, were eligible for further processing and analysis. A summary of the patient data is shown in Table 1.

**Table 1:** Patient coding and demographic data. *UKW* University Hospital Würzburg, *UKM* University Hospital Marburg

Patient ID	Location	Age	Gender	Patient ID	Location	Age	Gender
IP1	UKW	44.7	Female	MIP1	UKM	20.7	Female
IP2	UKW	65.3	Female	MIP2	UKM	25.9	Male
IP3	UKW	24.9	Female	MIP3	UKM	57.9	Female
IP4	UKW	28.6	Female	MIP4	UKM	36.6	Male
IP5	UKW	55.2	Female	MIP5	UKM	55.3	Male
IP6	UKW	21.0	Female	MIP6	UKM	67.0	Male
IP7	UKW	54.7	Female	MIP7	UKM	58.2	Female
IP8	UKW	48.8	Female	MIP8	UKM	33.6	Female
IP9	UKW	45.1	Female	MIP9	UKM	36.7	Female
IP10	UKW	44.6	Female	MIP10	UKM	37.1	Female
IP11	UKW	59.9	Male	MIP11	UKM	57.9	Female
IP12	UKW	26.2	Female	MIP12	UKM	31.1	Female
IP13	UKW	36.3	Male	MIP13	UKM	35.6	Male
IP14	UKW	38.9	Female	WIP1	UKW	34.0	Female
IP15	UKW	54.8	Male	WIP2	UKW	61.0	Female
IP16	UKW	63.8	Female				
IP17	UKW	19.8	Male				
IP18	UKW	59.2	Female				

The mean age of the UKW patients participating in the study was 44 ± 15 years and of the UKM patients 43 ± 15 years. The age difference between the two groups was statistically not significant. Nine male patients and 24 female patients were enrolled in the study (see Table 1). Due to technical difficulties with blood preparation and foci counting, the data of patient MIP3 had to be excluded from further analysis.

### Absorbed dose, DNA damage and repair

**Fig. 1:**
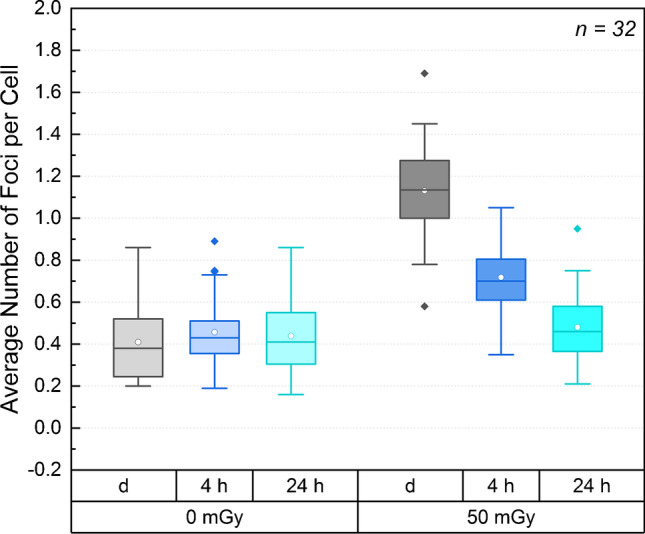
Box plot of the average number of foci per cell of all patient samples before and after ex vivo irradiation. The respective nominal time differences after irradiation and absorbed doses are shown in the bottom of the figure (d = directly fixed, i.e. 0 h repair time). The box comprises the 2nd and 3rd quartile of the data, the horizontal line defines the median and the circle the mean. Outliers (> 1.5 × interquartile range (IQR) are marked by filled diamonds

Raw data on the number of foci per cell and on the absorbed doses for each patient are provided in Table 2. Figure 1 shows, as boxplot, the average number of foci per cell before and after irradiation with [^131^I]NaI. The respective nominal absorbed doses and time difference after irradiation are shown in the bottom of Fig. 1.

**Table 2:** Raw data of the average number of foci per cell values and RIF repair rates for all patient data analysed. *SD* standard deviation, *NC* no convergence of the fit

	Average number of foci per cell	Average number of foci per cell	Average number of foci per cell	Average number of foci per cell	Average number of foci per cell	Average number of foci per cell	Repair rate R (h^−1^)	SD Repair rate (h^−1^)
Absorbed dose to the blood	0 mGy	0 mGy	0 mGy	50 mGy	50 mGy	50 mGy		
Repair time	0 h	4 h	24 h	0 h	4 h	24 h		
IP1	0.22	0.19	0.20	0.82	0.46	0.24	0.19	0.04
IP2	0.23	0.31	0.29	0.83	0.59	0.29	0.19	0.01
IP3	0.22	0.21	0.26	0.58	0.35	0.34	0.23	0.15
IP4	0.20	0.22	0.16	0.94	0.62	0.23	0.12	0.03
IP5	0.38	0.46	0.35	0.92	0.70	0.47	0.10	0.08
IP6	0.31	0.36	0.30	1.05	0.49	0.45	0.43	0.32
IP7	0.66	0.65	0.86	1.69	1.05	0.68	0.24	0.13
IP8	0.75	0.62	0.63	1.15	0.80	0.75	0.06	0.06
IP9	0.44	0.47	0.49	0.93	0.72	0.53	0.16	0.03
IP10	0.38	0.41	0.37	1.12	0.60	0.42	0.34	0.08
IP11	0.86	0.73	0.69	1.31	0.81	0.95	NC	
IP12	0.37	0.43	0.50	1.22	0.80	0.45	0.21	0.05
IP13	0.56	0.75	0.64	1.24	0.92	0.62	0.35	0.04
IP14	0.45	0.35	0.34	1.38	0.66	0.42	0.27	0.08
IP15	0.21	0.35	0.29	0.78	0.60	0.36	0.19	0.08
IP16	0.63	0.75	0.61	1.21	1.04	0.59	0.18	0.03
IP17	0.43	0.36	0.31	1.21	0.70	0.44	0.18	0.11
IP18	0.43	0.45	0.41	1.12	0.74	0.52	0.20	0.11
WIP1	0.31	0.49	0.47	1.11	0.74	0.54	0.29	0.07
WIP2	0.60	0.43	0.58	1.33	0.84	0.48	0.17	0.10
MIP1	0.21	0.32	0.30	1.05	0.67	0.37	0.21	0.06
MIP2	0.34	0.48	0.50	1.32	0.65	0.53	0.44	0.05
MIP4	0.39	0.38	0.44	1.14	0.74	0.50	0.17	0.04
MIP5	0.50	0.61	0.53	1.31	0.92	0.63	0.23	0.10
MIP6	0.45	0.49	0.55	1.21	0.83	0.59	0.20	0.03
MIP7	0.66	0.89	0.64	1.45	1.03	0.70	0.43	0.13
MIP8	0.54	0.51	0.55	1.32	0.68	0.57	0.38	0.03
MIP9	0.24	0.39	0.32	1.13	0.58	0.21	0.39	0.20
MIP10	0.26	0.40	0.41	1.16	0.60	0.32	0.38	0.14
MIP11	0.38	0.51	0.35	1.11	0.72	0.44	0.31	0.14
MIP12	0.25	0.39	0.4	0.95	0.65	0.36	0.25	0.05
MIP13	0.24	0.26	0.29	1.11	0.68	0.38	0.16	0.06

**Fig. 2:**
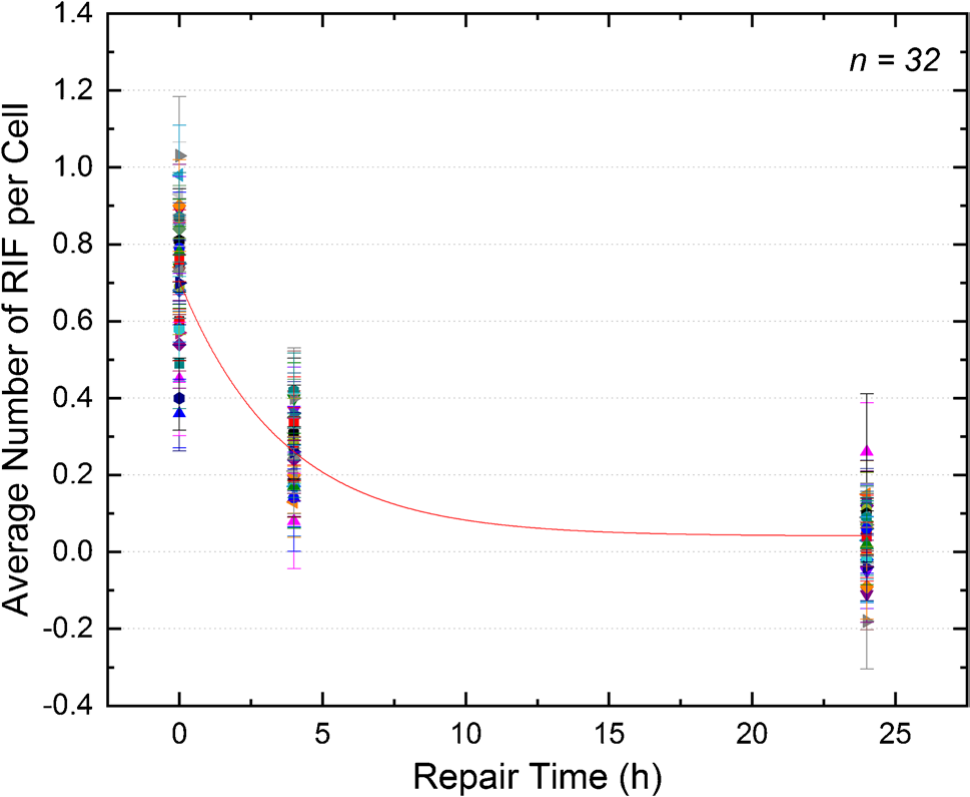
Average number of RIF per cell as a function of the repair time. The symbols denote the individual patients (*n* = 32). Red curve: population-based fit function according to Eq. 1

The mean absorbed dose in all samples irradiated ex vivo was 50.1 ± 2.3 mGy. The number of observed foci per cell for the baseline samples ranged from 0.16 to 0.86 foci per cell, after irradiation for 1 h from 0.58 to 1.69 foci per cell. In Fig. 1, a boxplot of all measured foci values before and after irradiation with [^131^I]NaI is shown. The values at all time points except the baseline samples at *t* = 0 h were distributed according to a normal distribution. The median of the number of foci per cell at *t* = 0 h (0-d) was 0.38 (min: 0.20, max: 0.86) and the mean number of foci per cell was 0.46 ± 0.17 (0-4 h), and 0.44 ± 0.16 (0-24 h) for the baseline values. After irradiation, the mean values were 1.13 ± 0.22 (50 mGy-d), 0.72 ± 0.16 (50 mGy-4 h) and 0.48 ± 0.16 (50 mGy-24 h) foci per cell. Except for 0-d and 0-4 h, the baseline average number of foci per cell was statistically not different.

The average number of foci per cell of the irradiated samples was significantly higher than the baseline values at all time points when compared to baseline. This was the case even for *t* = 24 h (*p* < 0.0059). For all irradiated samples (50 mGy-d, 50 mGy-4 h, 50 mGy-24 h), there was a significant difference of the average numbers of foci per cell.

The mean of the average number of RIF per cell after irradiation with a nominal absorbed dose of 50 mGy was 0.72 ± 0.16. This value is, within the respective uncertainties, in good agreement with the ex vivo calibration data calculated with the calibration curve provided by Eberlein et al. (expected value: (0.77 ± 0.03) RIF per cell) [14].

At *t* = 4 h, the mean value of the average number of RIF per cell was 0.26 ± 0.09, and at *t* = 24 h, 0.04 ± 0.09.

A monoexponential fit (Eq. 1) was performed for each patient individually. The fraction of unrepaired RIF per cell Q was set to 0, as, otherwise, a monoexponential fit comprising three data points per patient is not meaningful. Table 2 provides the individual RIF repair rates *R* according to Eq. 1 except for patient IP11 for whom the fit did not converge. The resulting values of *R* follow a normal distribution. The mean value of the individual fits is 0.25 ± 0.10 h^−1^. A monoexponential fit, comprising the three mean values of the average RIF per cell for each time point, results in a decay rate of 0.25 ± 0.05 h^−1^. Both values agree within their respective uncertainties.

For deriving the fraction for unrepaired foci, a combined fit through all data points (including all individual RIF values of all patients) was performed according to Eq. 1. In this case, *N*_0_ was 0.71 ± 0.02 RIF per cell, the repair rate *R* was 0.28 ± 0.03 h^−1^, and the value for *Q* was 0.06 ± 0.02 RIF per cell. This observation of Q > 0 is in agreement with our finding of a statistically significant elevated average number of foci per cell of the irradiated samples at *t* = 24 h (50 mGy-24 h, see Fig. 2). *Q* corresponds to about 6% unrepaired RIF per cell after irradiation.

## Discussion

This study provides a systematic approach to characterize DNA damage induction and repair of ^131^I-internally irradiated blood samples of patients before their first radioiodine therapy. In this approach, we closely mimicked ex vivo the irradiation of patient blood in the first hours after radioiodine therapy and investigated the DSB repair over the following 24 h.

As has been pointed out in a theoretical study by Dale and Fowler [31], that, if the rate of repair at any instant is directly proportional to the number of unrepaired lesions remaining (first-order process), the sublethal DNA damage is expected to be repaired monoexponentially over time. However, the same authors also observed that monoexponential repair models do not fully explain the results of several clinical studies. These data show a slowing down of the repair rate over time. According to Dale and Fowler, the DNA damage repair is very often described by an empirical linear combination of two or more monoexponential repair processes [31]. This is in line with studies of the DNA damage focus assay by Horn et al. [21] and Mariotti et al. [22], who discovered that the number of RIF per cell decreases over time with the onset of DNA repair and follows a biexponential function.

A monoexponential fit of the mean values of our data provided a decay rate of 0.25 ± 0.05 h^−1^. In comparison, the decay rate of the fast in vivo repair rate provided by the model calculations in 20 patients after radioiodine therapy by Eberlein et al. [3] of 0.33 ± 0.13 h^−1^ is similar as the ex vivo values obtained in this work. However, a qualitative comparison of the data shows a delay in the in vivo repair of the patients’ RIF [3] compared to the ex vivo study presented here. The underlying reason is, most likely, the continuous irradiation at low-dose rates in the patients even at late time points (e.g. see Lassmann et al. [13] and Eberlein et al. [3]).

In this study, *R* = (0.25 ± 0.10) h^−1^ was obtained as the mean value of the individual fits for the RIF decay rate. This value is slightly lower than the values calculated from the data of Löbrich et al. (23 patients, Fig. 5 of [18]) of 0.29 h^−1^ and 0.35 h^−1^ for lymphocytes, irradiated externally with X-rays resulting in absorbed doses of 20 mGy and 100 mGy, respectively [18]. It is also lower compared to the value determined by Horn et al. of 0.3495 h^−1^ for absorbed doses ≥ 0.5 Gy (data of 21 healthy donors) [21]. Beels et al. reported, for three volunteers whose samples were irradiated with 0.2 Gy X-rays and γ-rays, a value for the decay rate of 0.25–0.29 h^−1^ [19]. This value is in the same range as the results provided in this study. Our results are also in agreement with the value of 0.23 h^−1^ based on the cell culture data on fibroblasts by Mariotti et al. at higher absorbed doses (1–2 Gy) [22]. Yin et al. observed, after irradiating blood samples of 11 patients with absorbed doses of 0.5 Gy and more, a repair rate of 0.277 ± 0.014 h^−1^ for baseline and 0.293 ± 0.011 h^−1^ for blood samples taken 1 h after irradiation [25], a value that is also in close agreement with our findings. Overall, comparison with these studies, all of which used X- or γ-rays for external irradiation, shows that the progression of DNA repair after internal irradiation with radionuclides is very similar ex vivo, despite the differences in absorbed doses and dose rates.

One limitation of this study is that we had to restrict our experiments to three time points ≤ 24 h after irradiation (0 h, 4 h, 24 h) due to technical constraints. Therefore, the longer-lived component of the decay observed, e.g. by Horn et al. [21], could not be reproduced in this work. However, we still see an elevated mean number of RIF per cell 24 h after irradiation, which is in line with the observations of other studies on irradiated PBMCs summarized by the review of Siddiqui et al. [32].

A second limitation is that only one absorbed dose was studied due to the limited amount of blood that could be drawn from the patients. Thus, a possible dose dependency of DNA repair could not be analysed. Grudzenski et al. [20] (absorbed doses between 2.5 and 200 mGy) and Lengert et al. [24] (absorbed doses between 12 mGy and 1 Gy) observed a reduced repair efficiency after irradiation with low absorbed doses (≤ 20 mGy) in their cell studies. In both studies [20, 24], the samples were externally irradiated with dose rates larger than 1.8 Gy h^−1^, much larger as compared to our study. As we irradiated the samples with a fixed absorbed dose of 50 mGy with a low-dose rate of 50 mGy h^−1^ by internal irradiation, the dependency of the DNA damage and repair on the absorbed dose and dose rate, induced by radionuclides after internal irradiation, needs to be studied in the future. The dose rate 50 mGy h^−1^ was chosen to simulate the dose rate in patients in the first hours after radioiodine therapy [3]. Another reason why the irradiation time of 1 h was chosen was to maintain comparability with previous studies [4, 14].

Another limitation of this study is that the behaviour in vivo could not be entirely replicated by ex vivo experiments. By adding [^131^I]NaI solution to patient blood, we were able to simulate DNA damage induction in the patient during the first hours after radioiodine administration. However, the crosstalk between DSB induction and repair observed in vivo, due to the decreasing but still considerable dose rate at later time points, cannot be directly simulated ex vivo. Additionally, ex vivo settings rarely mimic the systemic antioxidant capacity that is present in in vivo irradiation experiments. To work out the difference to the situation in the patients more precisely, a comparison with matching in vivo data and clinical parameters is necessary, which has to be addressed in future studies. This analysis could contribute to a better understanding of inter-patient variability in DNA repair and facilitate individualized treatment planning in nuclear medicine in the future.

## Conclusion

Our study in a large patient cohort provides novel data on DNA damage repair in PBMCs after internal ex vivo irradiation of patients’ blood samples before radionuclide therapy. Overall, our data show that the ex vivo repair after an internal irradiation with low-dose rates follows similar patterns noted after external irradiation with high-dose rates. DNA damage is almost completely repaired after 24 h, with a repair rate comparable to that of external irradiation with γ- or X-rays.
